# The Contribution of Dairy Bedding and Silage to the Dissemination of Genes Coding for Antimicrobial Resistance: A Narrative Review

**DOI:** 10.3390/antibiotics13090905

**Published:** 2024-09-22

**Authors:** Armin Tarrah, Dong Zhang, Pariya Darvishzadeh, Gisèle LaPointe

**Affiliations:** Dairy at Guelph, Canadian Research Institute for Food Safety, Department of Food Science, University of Guelph, Guelph, ON N1G 2W1, Canada

**Keywords:** antimicrobial resistance genes, horizontal gene transfer, mastitis, milk quality

## Abstract

Antimicrobial resistance (AMR) is a concern in the dairy industry. Recent studies have indicated that bedding serves as a reservoir for antimicrobial-resistant bacteria and antimicrobial-resistance genes (ARGs), while silage has been proposed as another possible source. The impact of AMR in dairy farming can be significant, resulting in decreased productivity and economic losses for farmers. Several studies have highlighted the safety implications of AMR bacteria and genes in bedding and silage, emphasizing the need for further research on how housing, bedding, and silage management affect AMR in farm environments. Exposure to sub-lethal concentrations of antibiotics, such as those from contaminated bedding and silage, can prompt bacteria to develop resistance mechanisms. Thus, even if antimicrobial usage is diminished, ARGs may be maintained in the dairy farm environment. By implementing proactive measures to tackle AMR in dairy farming, we can take steps to preserve the health and productivity of dairy cattle while also protecting public health. This involves addressing the prudent use of antibiotics during production and promoting animal welfare, hygiene, and management practices in bedding and farm environments to minimize the risk of AMR development and spread. This narrative review compiles the growing research, positioning the contribution of bedding and silage to the prevalence and dissemination of AMR, which can elicit insights for researchers and policymakers.

## 1. Introduction

The indiscriminate use of antibiotics in animal husbandry has contributed to the emergence of antimicrobial resistance (AMR) in bacteria and the emergence of multidrug-resistant microorganisms worldwide [1,2]. AMR occurs when microorganisms develop the ability to survive exposure to antimicrobial drugs that were previously effective against them [3]. It is estimated that the annual antimicrobial usage in animal husbandry will reach over 200,000 tons by 2030 [4]. However, measures are being implemented to reduce antimicrobial use (AMU) on farms. While nontherapeutic usage of antibiotics for disease prevention and growth promotion is banned in many developed countries, therapeutic use of antibiotics remains prevalent [5]. This therapeutic use, driven by the need to treat infections, still introduces significant volumes of antibiotics into the dairy environment. Consequently, even therapeutic use can lead to the selection of antimicrobial-resistant bacteria carrying ARGs [6,7], as these genes provide a survival advantage under the selective pressure of antibiotic exposure. In addition, the release of unmetabolized antibiotics or their residues into the environment (feces, manure, and effluents) or by feeding waste milk from antibiotic-treated cattle to young animals has increased the dissemination of antimicrobial-resistant bacteria and ARGs [8,9]. These residues could persist in the environment, where they continue to exert selective pressure on microbial communities and also contribute to the accumulation of resistant bacteria in farm settings. Over time, this can lead to the contamination of soil and water sources, further spreading AMR [8,9].

Horizontal gene transfer (HGT) mechanisms play a role in the dissemination of ARGs within natural ecosystems [10,11]. There are three primary mechanisms of HGT, namely DNA transformation, conjugation, and transduction. Transformation involves bacteria taking up naked DNA from the environment. Conjugation is typically a plasmid-mediated process occurring between a donor and a recipient bacterium, and transduction is linked to bacterial phage DNA transfer through infection. All three HGT mechanisms have the potential to propagate ARGs throughout the microbial communities in the dairy production environment [10,11]. ARGs transmitted to human-associated bacteria may lead to infections that are difficult to treat [12,13]. Dairy cattle that are infected with antimicrobial-resistant bacteria may experience prolonged illness, reduced milk production, and even death, as there may be limited options for effective treatment [14].

Recently, there have been reports on detecting antimicrobial-resistant bacteria and ARGs in dairy farm environments [15,16,17,18,19]. Free and intracellular ARGs from dairy environments can be found in raw and pasteurized commercial milk, thereby posing an exposure risk to humans through the consumption of dairy products [12,20]. Beyond this, there is also an exposure risk associated with close contact between humans and animals, particularly for farm workers or others who regularly interact with cattle. If cattle are infected with resistant bacteria, the risk extends to meat consumption as well, as these resistant bacteria can potentially be transmitted to humans through the food chain. While the exact risk level is still being studied and is likely quite low, it remains a concern due to the potential for the expansion of bacteria that are resistant to antibiotics [21]. Several studies have collectively emphasized the critical gaps in evidence within the field of AMR, particularly in the contexts of environmental, animal, and feed-related transmission pathways, highlighting the pressing need for more data to improve the assessment of risk. Additionally, there is a lack of knowledge on how antimicrobial-resistant bacteria survive digestion in the stomach and pass to the colon, which further complicates the assessment of risk. These investigations underscore the complexity of addressing significant knowledge voids to guide future research endeavors in the field of AMR [22,23,24,25].

Organic bedding materials, such as straw and wood shavings, support a more diverse microbial community with higher bacterial loads, including both beneficial and potentially harmful bacteria, due to their organic content and moisture absorption, which could potentially lead to the colonization of the udder [26]. In contrast, inorganic bedding, such as sand, maintains a lower microbial diversity and reduced bacterial load, but poses other challenges for management. These differences indicate a clear relationship between the bedding material type and the microbial communities they support, which can have important implications for cow health, milk quality, and overall farm hygiene. Bacteria from contaminated bedding can enter the udder and cause infections, such as mastitis, which is a major cause of reduced milk production, high veterinary costs, and animal welfare concerns in dairy farming [27,28]. In contrast, high-quality bedding materials, combined with appropriate management practices, can provide a dry and comfortable environment that helps prevent bacterial growth and reduce the risk of udder infections [27,28]. Clean and dry bedding is important for maintaining the well-being of dairy cattle, given their daily exposure [28,29,30].

Silage is one of the essential components of cow feed in North America. Silage is a type of fodder that can be made from maize, sorghum, cereals, legume plants, or grasses by the process of anaerobic fermentation and could contribute to animal health directly as each animal consumes an average of 25–27 kg of silage per day [31]. Therefore, the assessment of the dairy bedding and silage, in terms of their microbial community, ARGs, and their potential threats, would be an important step in providing recommendations for the best farm management practices.

In this narrative review, our primary objective is to present recent progress on the prevalence and variability of ARGs and antimicrobial-resistant bacteria in bedding and silage, explore their possible transmission pathways, and identify critical research gaps. The choice of a narrative review approach is driven by the limited number of studies and the diverse nature of the available literature on these specific topics within dairy environments. A narrative review allows us to synthesize the available evidence, providing a comprehensive overview and analysis of the diverse perspectives in the field. Additionally, we compile the practical measures suggested by studies to mitigate the prevalence of ARGs and antimicrobial-resistant bacteria in dairy environments.

## 2. Literature Review Methods

To identify pertinent studies, a search was conducted using four databases accessed through the following interfaces: Web of Science (covering three databases: the Science Citation Index Expanded (SCI-EXPANDED), the Emerging Sources Citation Index (ESCI), and the Conference Proceedings Citation Index—Science (CPCI-S)) and Scopus using the following Boolean search term: (“antimicrobial resistance” OR “AMR”) AND (genes OR bacteria) AND dairy AND (bedding OR silage). No restrictions were placed on the date of publication.

**Figure 1:**
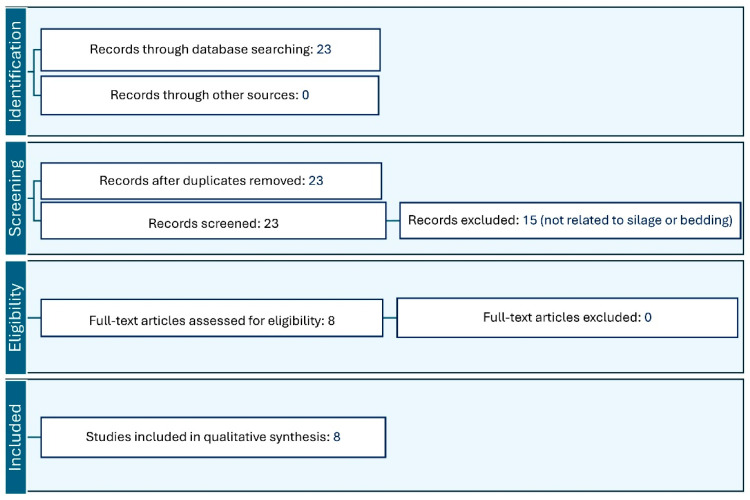
Flowchart of the database search and screening process.

Our inclusion criteria involved only studies published in peer-reviewed journals and focused on dairy. We prioritized articles that presented primary research findings, and reviews related to AMR in bedding and silage. The initial search aimed to identify a broad range of the literature, which was subsequently refined through a systematic screening process. Two reviewers independently screened the titles and abstracts of the articles to identify relevant studies. Articles that passed the initial screening were then assessed in full text by both reviewers. In cases where there were grey areas, particularly when studies were related to milk but did not focus on silage and bedding or did not analyze silage or bedding, the reviewers reached a consensus, ensuring that only studies meeting the inclusion criteria were selected. The final selection was guided by the relevance of the studies to the primary objectives of this narrative review (Figure 1).

## 3. Bedding for Dairy Cattle and AMR Prevalence

Providing appropriate bedding is essential for dairy cattle, as they spend 8–16 h/d lying down [28,29,30]. The choice of organic bedding material, such as wood shavings, sawdust, straw, and recycled manure solids, versus inorganic bedding, such as sand, results in distinct challenges for preserving udder health, hygiene, and milk quality [32,33]. The existence of pathogenic bacteria and ARGs in dairy environments, including in bedding, should be among the critical concerns related to dairy product safety and consumer health, as they might be transferred to dairy products. Bedding can serve as a reservoir for antimicrobial-resistant bacteria and genes, which can then contaminate raw milk through direct or indirect contact with dairy cattle. Determining the presence of multiple ARGs in raw milk could be an indicator of the potential prevalence of these genes in the dairy environment [12]. This section is organized to first present the current understanding of the types of AMR markers and their host bacteria commonly found in bedding, as well as how these compare to those found in manure and manure-derived bedding materials. Given that many pertinent studies compare multiple bedding types, the remainder of the section is structured around the properties of organic versus inorganic bedding.

According to the available literature, the most frequently reported types of AMR markers found in bedding include genes coding for beta-lactamases, aminoglycoside-modifying enzymes, sulfonamide-resistant dihydropteroate synthase, carbapenemase, tetracycline, and macrolide resistance (Table 1). The genes encoding resistance to these antibiotic classes have been detected in bacteria, such as *Escherichia coli*, *Bacillus cereus*, *Klebsiella pneumoniae*, *Listeria monocytogenes*, and *Salmonella enterica*, in the dairy environments [18,34,35,36,37]. These bacteria can cause illness in cattle, and some of them are critical foodborne pathogens that impose a burden on public health when transmitted to humans through milk consumption.

It has been found in a study of calves on 20 dairy farms [8] that the used bedding contained multidrug-resistant *E. coli* isolates with the same resistance pattern as the calves from the same farm. In another study, calves were shown to acquire cephalosporin-resistant *E. coli* from used bedding [9]. The urine from calves treated with cephalosporins was shown to contribute to the selection for ceftiofur-resistant *E. coli* in the soil [9]. The intricate interplay between antibiotic use, soil-borne organisms with natural antimicrobial production, and the selection of resistant markers requires further investigation to elucidate the dynamics involved. In dairy cows, antibiotics are primarily used therapeutically to address mastitis during lactation and the drying-off stage [40,41]. However, in the past, on numerous farms, antibiotics were not only used for therapeutic purposes but also disease prevention. Allowance for preventive use varies by country and has been banned in some places, such as the European Union [42]. In addition to antibiotics, chemical disinfectants and heavy metal compounds, such as chlorhexidine and copper sulfate, are commonly used in dairy farming [43]. Research suggests that these compounds may contribute to the co-selection of resistance mechanisms, where genes conferring resistance to one type of antimicrobial also confer resistance to others [44,45]. This co-selection phenomenon, documented in microbial populations, underscores the need for further investigation into the implications for AMR spread in the contexts of bedding and silage [44,45].

While studies have investigated the prevalence of bacteria in manure and its treated by-products, it is important to recognize that the survival of resistant bacteria and free ARGs in bedding differs from their prevalence in manure. The prevalence of bacteria in manure is largely influenced by the conditions within the gut environment. In contrast, the survival of bacteria and ARGs in bedding materials is affected by environmental factors, such as temperature, humidity, the presence of competing microorganisms, and the type and management of the bedding material. This distinction is particularly relevant for organic bedding materials, such as recycled manure solids (RMS), which are derived from treated manure and are often utilized as a bedding option due to its availability and economic advantages [46]. Composting has been shown to effectively reduce the concentration of ARGs, including tetracycline-resistance genes, in manure [47]. This process can mitigate risk to significantly enhance the safety of recycled solids from manure as a bedding option, as it promotes the degradation of organic matter and the destruction of pathogenic bacteria and ARGs [47].

A recent study in China indicated that 44% of the *K. pneumoniae* isolated from dairy environments, including bedding, were multidrug-resistant (MDR) [19]. The most prevalent ARGs detected in this study were *blaTEM*, *blaSHV*, *strA*, *strB*, *aadA1*, and *aac(60)-Ib-cr*, coding for resistance against beta-lactams and aminoglycoside classes [19]. In another study investigating multidrug-resistant *Salmonella* in feces and environmental dairy samples, recycled sand bedding had the highest prevalence of *Salmonella* Newport among the environmental dairy samples [34]. All *Salmonella* Newport strains from recycled sand bedding were resistant to ampicillin, streptomycin, trimethoprim–sulfamethoxazole, chloramphenicol, and tetracycline [34]. Recycled sand utilized as bedding material has undergone reclamation processes for reuse. This distinction is crucial, as it influences the accumulation of organic matter and potential pathogen interactions [26]. Sand accumulates organic matter, such as manure, urine, and undigested feed, making it challenging to clean and remove pathogens effectively [48]. Additionally, unlike some bedding materials with natural antimicrobial properties, such as wood shavings with phenolic compounds, sand lacks inherent antibacterial characteristics [49]. The physical properties of sand, including its granular structure, may pose challenges in terms of cleaning and removing potential contaminants, such as antimicrobial-resistant bacteria, especially those that form biofilms [27]. An effective sand recycling process would involve thorough cleaning, perhaps including heat treatment to remove these contaminants. Heat treatment is not generally included in sand recycling processes. However, incorporating heat into the recycling process may enhance the removal of specific contaminants, especially those pathogens associated with biofilm production [50]. The use of heat in sand recycling is under development, emphasizing the need for further research to investigate the efficacy of treatment methods, such as bedding driers, and address challenges related to biofilm removal. If steps to reduce moisture and organic matter content are not effective, sand may continue to harbor and promote the retention of antimicrobial-resistant bacteria. A study by Rowbotham and Ruegg (2016) [26] showed higher counts of Gram-negative bacteria from pre-milking teat swabs taken from cows on recycled sand (screw-type sand separator) compared to deep-bedded new sand. This underscores the importance of understanding and refining recycling processes to improve the microbial quality of reclaimed sand [26].

A study investigating milk quality reported significant bacterial populations in the bedding materials of three bedding types used in the UK, namely sand, sawdust, and RMS [51]. Among the zoonotic bacteria, *L. monocytogenes*, a Gram-positive bacterium, was especially prevalent in sand bedding, accounting for 58.5% of farms. Additionally, the highest count of *Staphylococcus* spp. was recorded on a farm using sand bedding, reaching 8 × 10^6^ (CFU/g). The elevated presence of both of these species in sand might be attributed to factors such as the treatment of the sand, potential soil cross-contamination, or less frequent replenishment of the bedding [51]. Lowering the moisture and organic matter content of sand bedding is thus crucial to mitigate these microbial risks [51]. In this study, sand exhibited 1 log more coliforms compared to sawdust. This observation suggests a potential correlation with AMR, as many Gram-negative bacteria, which include coliforms, carry ARGs [51]. This difference may be influenced by the frequency of replacement, as sawdust is typically refreshed more often than sand.

Multidrug-resistant *L. monocytogenes* have also been found in straw bedding [18]. The authors reported that 12.5% of straw-bedding samples were contaminated with multidrug-resistant *L. monocytogenes* strains that are resistant to imipenem, penicillin G, streptomycin, and sulfamethoxazole–trimethoprim. For straw bedding, the organic nutrient content and moisture absorption allow for the growth of most microbes [26], thus compelling frequent refreshing and replacement. Recent studies also highlight the importance of effective disinfection and regular management for controlling microbial contamination in organic bedding, such as straw [25,52]. Propionic acid and formaldehyde have proven effective in reducing microbial levels, with propionic acid showing better results against molds. Ozone treatment, however, offers a greener alternative, significantly reducing bacterial and fungal levels by over 1.5 logs (cfu/g) [25,52].

Bedding contamination and AMR risk can be related to feeding management in several ways. The management of animal feeding can impact the quality and composition of their manure, which in turn, can affect the microbial load in bedding and the risk of AMR transmission [53,54]. Overfeeding animals or an imbalanced diet will result in high levels of undigested feed and nutrients in animal manure, creating a favorable environment for bacterial growth in the bedding [53,54]. This may increase the risk of AMR transmission, as bacteria in the manure can spread to the bedding and potentially contaminate other areas, including the crops where manure is spread on the farm. Similarly, administering diets that contain antimicrobial agents such as antibiotics to animals can result in the excretion of these antibiotics into the manure [55,56,57], which can further lead to the emergence of AMR bacteria in the manure and the surrounding environment, including the bedding material. Moreover, poor or inadequate feed can lead to nutritional deficiencies, causing cattle to consume their contaminated bedding out of hunger or nutrient-seeking behavior. This can further exacerbate the spread of antimicrobial-resistant bacteria or their genes, as the ingestion of contaminated bedding directly introduces these pathogens into the animals’ systems, increasing the risk of infection and AMR dissemination.

On the other hand, the composition and diversity of bacterial taxa may contribute to the selection or mutation of intracellular ARGs, as some bacterial taxa are known to carry mobile genetic elements and ARGs more frequently than others.

The microbial composition of dairy environments, particularly in relation to bedding materials, contributes significantly to our understanding of AMR prevalence in raw milk. A recent study in China on the microbial composition of three types of bedding (sand, rice husk, and recycled manure solids) and the collected milk from cows housed on those bedding types confirmed the relation between the microbiota composition of bedding and that of milk [37]. This relationship highlights the potential for cross-contamination, where bacteria from the bedding can be transferred to the teats of cows and subsequently to the milk [37]. The cows bedded on recycled manure solids and sand had a significantly higher proportion of Bacillota (formerly known as Firmicutes) in their milk compared to the milk from cows on rice-husk bedding [37]. On the other hand, cows bedded on rice-husk bedding had a higher proportion of Pseudomonadota [37]. A higher prevalence of Bacillota, particularly in the recycled manure solids, might be indicative of a higher survival of potential mastitis pathogens, such as *Streptococcus uberis* and *Enterococcus faecalis* [58,59].

A higher prevalence of Pseudomonadota in rice-husk materials may suggest a higher abundance of certain bacteria, such as *E. coli*, *K. pneumoniae*, and *Pseudomonas aeruginosa* [37]. The reason for their increased presence in rice-husk bedding is currently unknown. The dominance of these bacteria, which can cause mastitis in cows under certain environmental conditions, can influence the overall microbial ecology of the bedding and potentially affect cow health. Moreover, Wu et al. (2022a) [37] reported that recycled manure solids might carry more pathogens, as recycled manure solids come from animal feces, which may contain potentially hazardous microorganisms if the treatment of manure does not eradicate them. However, the milk collected from the cows housed on recycled manure solids was found to have fewer ARGs than from cows on sand bedding. In contrast, milk collected from the cows housed on sand bedding with a lower abundance of bacteria was found to have a higher prevalence of ARGs (intracellular and free). This counterintuitive finding raises questions about the recycling processes for both materials. Unfortunately, the study did not assess the presence of ARGs and antimicrobial-resistant bacteria before and after the treatment. In the future, such comparisons would provide insights into the effectiveness of the recycling process for AMR mitigation. While the study of Wu et al. (2022a) [37] provides valuable insights into the AMR gene patterns in the milk collected from cows housed on different bedding materials, it is important to note that the effect of bedding type on AMR prevalence within the bedding material itself was not examined. To gain a comprehensive understanding of the AMR dynamics, future research could focus on investigating the intracellular and free AMR gene abundance and diversity directly within the bedding material, ensuring an accurate control of confounding factors.

Recent reports on the prevalence of microorganisms carrying ARGs in dairy bedding also highlight the importance of regular risk evaluation to assess the presence of these ARGs in bedding [19,37]. To evaluate the issue, further investigation is required that considers factors such as the stall and bedding type, since each type appears to have distinct bacterial habitats, populations, and prevalence of ARGs [37]. Furthermore, each type of bedding and housing may have specific management and cleaning practices that can influence the prevalence and transmission of AMR [60]. The development and propagation of AMR are favored when the ventilation or drainage is poor, which promotes bacterial growth [61,62]. Overcrowding animals or shared stalls will promote the transfer of antimicrobial-resistant bacteria among animals, thus increasing their prevalence in the herd [63]. Some of the risk-mitigation strategies for the reduction of antimicrobial-resistant bacteria in bedding essentially center around the same goals as reducing bacterial growth and disease dissemination, namely keeping bedding clean and dry, whether organic or inorganic.

Additionally, the compost bedded pack (CBP) housing system, which involves the use of a deep-bedded pack of organic materials such as sawdust or straw that is regularly aerated to facilitate composting and provide a comfortable bedding surface for cows, remains relatively unexplored in the context of AMR [64]. Investigating the bedding used in this housing system for AMR dynamics could provide data on the prevalence and transmission of antimicrobial-resistant bacteria and free ARGs in dairy environments, contributing to a more comprehensive understanding of microbial communities and resistance gene reservoirs. Factors such as variability in composting efficiency, the impact of stocking density, and the effects of tilling depth and frequency on microbial survival should also be considered in this investigation. Moreover, it is essential to investigate the difference between used and unused bedding. Used bedding contaminated with cow manure contains a higher number of microorganisms originating from the animal gut rather than from the bedding itself [37]. Future studies should consider the quality parameters of both used and unused bedding to understand their impact on microbial diversity.

## 4. AMR Prevalence in Silage

Over the past decade, researchers have been evaluating the importance of the prevalence of ARGs and the potential threats to the dairy industry [65,66,67,68]. However, few studies have investigated ARGs in silage, especially using advanced omics approaches (Table 1) [31,38]. Koutsoumanis et al. (2021) state that silage and other greens are a likely source and transmission route for AMR, but that information is still lacking [25].

Alfalfa silage, a perennial flowering plant in the legume family, is one of the most common roughages for dairy cows, which provides sufficient nutrients to improve milk quality and quantity [69]. A shotgun metagenomic study on alfalfa silage from China by Nagy et al. (2021) [31] reported the presence of *aadA2*, *ant(6)-Ia*, *ant(9)-Ia*, *aph(3’)-IIa*, *aph(3’)-IIIa*, and *dfrG*, which encode resistance to aminoglycoside and diaminopyrimidine. Interestingly, more than 50% of these ARGs (intracellular and free) were reported as mobile due to the presence of integrative mobile genetic elements, prophages, or plasmid-encoded genes within their sequences [31]. However, metagenomic sequencing has limitations in attributing the bacterial origin of genes due to complex microbial communities, incomplete reference databases, and horizontal gene transfer [70]. Therefore, the reported proportion of mobile ARGs should be interpreted with consideration of the limitations of the methods employed. The authors also reported *Weissella* (45.7%), *Pantoea* (18.5%), *Levilactobacillus* (13.5%), *Pediococcus* (6.7%), and *Lactiplantibacillus* (6.3%) as the predominant microbes [31]. While this information provides valuable insights into the microbial composition, further investigation is needed to assess any specific concerns related to these microorganisms, particularly in the context of AMR propagation.

An interesting study from China on alfalfa silage compared the presence of dominant intracellular and free ARGs in fresh (first cut) and ensiled alfalfa for the first time, illustrating the distribution of these ARGs [38]. The authors reported that the extended wilting period could increase AMR gene enrichment in fresh alfalfa. On the other hand, they concluded that using inoculant cultures, such as lactic acid bacteria (LAB), could reduce the prevalence of ARGs in ensiled alfalfa. More specifically, alfalfa silage inoculated with *L*. *plantarum* MTD/1 or *L*. *buchneri* 40788 reduced the abundance of total ARGs related to vancomycin, aminoglycoside, tetracycline, and fosmidomycin [38]. This reduction was achieved by lowering the number of host bacteria (bacteria that serve as hosts for ARGs) and limiting the enrichment of the ARGs located on plasmids. The host bacteria and their relevant ARGs mainly originated from *Pseudomonas*, *Pantoea*, *Enterococcus*, *Enterobacter*, *Staphylococcus*, *Lelliottia*, *Erwinia*, *Kluyvera*, and *Rahnella*, determined through metagenomic shotgun analysis [38]. The ARGs detected in alfalfa silage included *arlR*, *emrB-qacA*, *cpxR*, and *penA*. These genes primarily play roles in regulatory functions or other bacterial processes that may indirectly impact antibiotic resistance. In contrast, the genes reported by Nagy et al. (2021) [31] are directly associated with antibiotic resistance against specific classes of antibiotics. These genes can provide resistance to diaminopyrimidine and aminoglycosides (*aadA2*, *ant(6)-Ia*, *ant(9)-Ia*, *aph(3’)-IIa*, and *aph(3’)-IIIa*), as well as trimethoprim (*dfrG*).

Effective silage inoculants and maintaining the pH below 4.0 during silage fermentation prevent the emergence of antimicrobial-resistant bacteria and, thus, reduce ARG prevalence in silage, as a pH level higher than 4 can facilitate the growth of bacteria that may carry ARGs. Certain bacteria, such as *Clostridium* species, are known to be pH-sensitive [71]. In a study conducted by Dos Santos et al. (2022) [72,73], AMR was detected in the majority of *Clostridium* isolates, with most strains carrying plasmids that facilitated the mobilization of both virulence and AMR markers. While the presence of AMR in *C. perfringens* isolates was noted, the levels remained manageable. This highlights the importance of ongoing monitoring for AMR in *C. perfringens*, especially on farms where the dissemination of *Clostridium* isolates was observed. Emphasizing the utility of surveillance, particularly in feed samples, becomes beneficial for safeguarding animal health [72,73]. The prevalence of AMR is influenced by temperature, as a temperature range between 20 °C and 45 °C is favorable for the growth of mesophilic bacteria, many of which can carry ARGs [74,75]. Ensiling could be a feasible way to mitigate ARGs in forages. This reduction is believed to be achieved through mechanisms such as antimicrobial activity, pH reduction, and other factors associated with the fermentation process. However, a more in-depth exploration of the exact mechanisms and the effectiveness of additional LAB strains is warranted through further research.

Additionally, Zhang et al. (2022a) [15] reported that the addition of pyroligneous acid (PA) could improve fermentation quality, as well as reduce the AMR gene content of alfalfa silage, with 2% PA performing better than 1% PA. PA could reduce the relative abundance of ARGs (intracellular and free) by directly inhibiting potentially resistant bacteria [15]. Composting specifically applied to silage waste after the anaerobic digestion process (post-digestate composting) serves as an effective alternative management method for silage waste, aiming to remove both intracellular and free ARGs [76]. Notably, research using a quantitative polymerase chain reaction (qPCR) indicates an over 80% reduction in AMR gene abundance, determined by comparing gene copy numbers before and after composting [76]. Chemical additives and LAB in silage can both play vital roles in the prevalence and variability of ARGs. Wu et al. (2020) [39] reported that the microbial community associated with intracellular and free ARGs is present in the high-moisture corn-kernel silage. The microbial population was modified by adding vanillin, which led to an increase in the dry-matter content while decreasing the lactate and ammonia nitrogen content. Vanillin treatment increased the abundance of *Lactococcus lactis*, *Saccharomycodaceae*, *Hanseniaspora uvarum*, *Pseudomonas*, *Enterobacter cancerogenus*, and *Myviridae* [39]. The action of vanillin in silage seems to show selective antimicrobial properties, leading to an increase in specific beneficial microbes, such as *L. lactis*, and altering other microbial taxa [39]. The role might be primarily bacteriostatic, inhibiting the growth and proliferation of certain microbes while allowing or even promoting the growth of others [39]. The results also suggest that adding *L. brevis* to high-moisture corn-kernel silage can impact the intracellular transmission of AMR genes. This effect is likely due to acidification and pH reduction, which can inhibit the growth of certain bacteria commonly found in silage that may carry ARGs, such as *L. monocytogenes*, *Clostridium botulinum*, *B. cereus*, and *Enterobacteriaceae* [39]. Of the microbial species detected, *Pseudomonas* and *Enterobacter* are of particular concern in the context of AMR, as they have been associated with antibiotic resistance and may play a role in AMR gene transfer dynamics [77]. Further comprehensive studies are warranted to delve deeper into the mechanisms of the selective antimicrobial properties and the impact of vanillin on the dissemination of ARGs, offering a more nuanced understanding of the roles and implications of these species.

Thus, the results suggest that non-acid chemicals with antimicrobial properties could serve as useful silage additives, which provides some new information to improve silage additive selection and safety evaluations. Wu et al. (2020) [39] reported that the main bacterial genera in the silage were *Lactobacillus* and *Leuconostocaceae*, followed by *Saccharomycetes* and *Kazachstania,* which differs from what Zhang et al. (2022c) reported [78]. The differences between the findings of the studies might be attributable to the distinct nutrient composition of silage materials, as well as the inoculant strains used. For example, Zhang et al. (2022c) [78] used sorghum-stalk silage, while Wu et al. (2021) used sweet corn-kernel silage. Sweet corn-kernel silage has a higher crude protein content and lower NDF content than sorghum-stalk silage [79]. The difference in soluble carbohydrate content between the two types of silage materials plays an important role in shaping the microbial ecosystem. Sweet corn-kernel silage, containing higher crude protein and lower NDF, likely creates homofermentative conditions that favor the growth and metabolic activities of beneficial bacterial genera [75]. Therefore, the choice of silage material, combined with the specific inoculant, can significantly modify the microbial community composition and dynamics [75]. These findings underscore the importance of considering not only the nutrient composition but also the soluble carbohydrate content in the selection of appropriate inoculants for silage production. While understanding this concept is key for fermentation efficiency, it is equally imperative to consider the potential AMR implications. Changes in the microbial community could lead to an increased presence of antimicrobial-resistant bacteria, especially if certain microbes within the silage inherently carry acquired resistance genes. Such occurrences could potentially contribute to the dissemination of AMR. However, it is important to note that this hypothesis regarding gene transfer within silage environments and the potential connection to AMR dissemination requires further research for substantiation. To our knowledge, there is no direct evidence yet of gene transfer within silage environments. It is noteworthy that studies have assessed the spread of AMR in crops, providing valuable insights into the broader context of AMR dissemination, although they do not necessarily confirm the specific mechanisms within silage environments [80].

In conclusion, while the existing literature on AMR in silage has focused on alfalfa, it provides valuable insights into the dynamics of AMR in this specific type of silage. However, investigating AMR in other silage types, including grass silage, corn silage, and other forage crops, is necessary to understand a broader perspective and address potential variations in AMR profiles among crops.

## 5. Methodological Limitations and Considerations for Silage and Bedding

In recent investigations of AMR in raw milk, several studies have provided valuable insights into the prevalence and transferability of ARGs [12,20]. The study conducted in Budapest on raw milk samples highlighted the presence of ARGs encoding resistance to multiple antibiotics [12]. Specifically, the detection of PC1 beta-lactamase in the raw milk metagenome raised concerns about the potential transfer of resistance genes to bacteria, particularly those residing in the human gut [12]. Expanding our understanding, a study in the United States (California) employed a metagenomic approach, revealing AMR in all analyzed raw milk samples [20]. The identified ARGs conferred resistance to aminoglycosides, beta-lactams, and tetracyclines [20]. The demonstration of the active transfer of the *blaCMY-2* gene between bacterial strains emphasized the dynamic nature of AMR dissemination [20]. While these studies shed light on the prevalence and dynamics of AMR in raw milk, it is noteworthy that similar investigations in other dairy environments, particularly bedding and silage, remain limited. The use of advanced techniques, such as metagenomics, has proven invaluable for uncovering ARGs in raw milk samples, prompting the question of whether such methodologies could yield comparable insights for other aspects of the dairy farm. Establishing a correlation between ARGs and specific dairy environments, including bedding and silage, holds great potential for understanding the broader landscape of AMR in dairy farming.

Recognizing the limitations of traditional culture-dependent methods, particularly in capturing viable but non-culturable cells (VBNC), researchers have turned to advanced molecular techniques [81]. With the emergence of molecular techniques, including metagenetic 16S rRNA gene amplicon sequencing and metagenomic shotgun sequencing, we can extract valuable information about the genetic composition and ARGs in the microbial community of bedding and silage [70]. Metagenetic 16S rRNA gene amplicon sequencing provides taxonomic information about the bacterial and archaeal groups present in silage but does not reliably resolve the taxonomy below the genus level [70]. On the other hand, metagenomics shotgun sequencing captures DNA from all microbes, including bacteria, fungi, and viruses, offering a comprehensive view of the genetic potential and allowing for the detection of ARGs across multiple microbial phyla. It is important to note that these techniques do not differentiate between dead and live cells, as the total DNA extracted from the microbial community is analyzed [31,82]. Therefore, complementary methods, such as propidium monoazide (PMA) treatment, which involves a dye that selectively binds to and inhibits the DNA from dead or permeable cells, could enhance the accuracy of AMR gene detection in future studies on the silage microbiome. This treatment helps to distinguish between viable and non-viable cells by preventing the DNA of dead cells from being amplified in molecular analyses [83]. Moreover, molecular techniques may have limitations in detecting phenotypic resistance, as resistance can occur in strains without known resistance genes. Emphasizing novel approaches, such as omics technologies, can significantly enhance our understanding of microbial communities and their genetic potential for AMR [31]. Integrating advanced bioinformatic and machine-learning analytical techniques with omics data can yield deeper insights and patterns that can complement traditional methods [84]. This integration aims to extract meaningful patterns, correlations, and predictive models from high-dimensional omics data, such as genomics, metagenomics, or transcriptomics [84]. For instance, machine-learning models could be trained to predict ARG abundance based on metagenomic data, aiding in understanding the factors influencing AMR dynamics in the bedding and silage environments. By integrating diverse methodologies, the next step in AMR research would be establishing a more comprehensive framework that enables a holistic understanding of AMR dynamics in bedding and silage, paving the way for targeted intervention strategies and improved management practices.

Additionally, techniques such as nuclear magnetic resonance (NMR) for metabolite analysis offer unique insights into the impact of antibiotics in the context of silage [85]. Even though AMR mechanisms are often protein-based, the downstream effects of resistance can manifest in altered metabolic profiles [85]. By using NMR to profile these metabolites, researchers can gain a deeper understanding of bacterial adaptations at the metabolic level, thereby complementing the genomics or proteomics data and offering a holistic view of microbial responses in silage [86].

On the other hand, the challenges associated with reporting antimicrobial susceptibility data for environmental organisms within dairy systems should be recognized and addressed. For many soil-borne organisms, established breakpoints are lacking, posing a significant hurdle to the accurate interpretation of susceptibility results [87]. Furthermore, the intricate interplay of antibiotic producers among soil-borne organisms adds another layer of complexity to the dynamics of AMR in dairy environments. These producers can contribute to the maintenance of resistance genes, influencing the overall resistance landscape on farms [87].

In light of these challenges, a comprehensive monitoring program for dairy environments becomes indispensable. This program should not only investigate factors such as the selection of appropriate target bacterial strains containing ARGs but also delve into the nuances of reporting antimicrobial susceptibility data. Rigorous methodologies, including well-defined breakpoints for soil-borne organisms, need to be established to enhance the reliability of surveillance efforts. While significant progress has been made in AMR surveillance, acknowledging and addressing these challenges will further fortify our efforts in combating the global AMR crisis.

## 6. AMR Dissemination and Transmission Pathways

**Figure 2:**
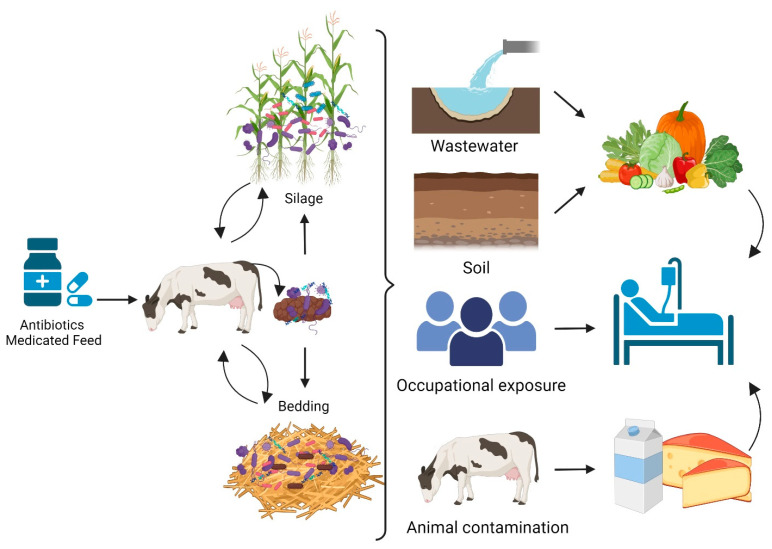
Potential transfer pathways of ARGs from contaminated bedding and silage to humans. Horizontal gene transfer (HGT) could occur among antimicrobial-resistant bacteria and indigenous bacteria of all environments indicated in the figure (Created with BioRender.com).

Free and intracellular ARGs can enter the animal gut by consuming water or contaminated feed and ultimately be released via excreted feces into the farm environment, including bedding materials [88]. ARGs from silage and bedding could be transferred directly to humans through occupational exposure at the farm level or indirectly through contaminated soil and wastewater at animal production facilities (Figure 2) [89]. In addition, these genes might be transferred by pathogens (intracellular ARGs), either directly from bedding or from the milking system to the animal teats and udder to finally find their way into milk, as well as being transferred through the milking system to other cows [90,91]. The risk of transfer to humans would then depend on the processing steps and the type of dairy product consumed [92]. While pasteurization is effective in reducing the risk of illness caused by harmful bacteria in milk, some consumers prefer to consume raw milk and raw milk products. Outbreaks related to unpasteurized dairy products have been linked to multiple types of microorganisms, such as *Campylobacter* spp., *L. monocytogenes*, *E. coli*, *Streptococcus bovis*, *Brucella* spp., *Salmonella* spp., and other enteric pathogens. Additionally, some thermoduric microorganisms, such as *B. cereus,* can survive the pasteurization process [93,94,95].

In the context of AMR transmission pathways to humans, it is noteworthy that various studies demonstrate the significant impact of sub-minimum inhibitory concentration (sub-MIC) levels of tetracyclines on bacterial resistance [96]. These studies, conducted on both defined single species and complex microbial communities, reveal a range of effects, including the selection for newly acquired resistance, enrichment of pre-existing resistance, alterations in bacterial community composition, heightened HGT, and increased bacterial virulence. These effects collectively introduce the potential to contribute to an escalation in the number of resistance genes, representing the fraction of bacteria that is resistant, and an elevation in the level of resistance, as denoted by the MIC value of the resistant cells within a microbial population [96].

ARGs in soil that has been contaminated with bedding materials, such as recycled manure solids, can be transmitted to humans through contaminated foods, such as vegetables and fruits, when crops have been treated with insufficiently composted manure [16]. Recent studies reported that soil samples contaminated with animal manure had significantly more free and intracellular ARGs related to the resistance of antibiotics, such as sulphonamide and tetracycline [97,98]. Moreover, ARGs have long-term persistence in soil and could remain for over 4 months [1,99,100]. Some bacterial taxa have a greater contribution to disseminating ARGs in the soil. Studies have investigated the connection between the persistence of ARGs and the diversity of microbial community members in manure-treated soil, and they reported that *Fluviicola*, *Flavobacterium*, *Clostridium*, *Leucobacter*, *Aquamicrobium*, *Pedobacter*, *Cellvibrio*, *Gelidibacter*, *Psychrobacter*, *Turicibacter*, *Pseudomonas,* and *Acinetobacter* were the genera involved in ARGs enrichment in manure-contaminated soils [100,101]. Contrary to the abovementioned studies, a recent shotgun sequencing study on the microbial composition of manure and fertilized field soil (corn and soybean field soil that received the same manure for fertilization) of 15 dairy farms in the USA indicated that microbial community composition in field-soil communities was distinct from those in manure regarding microbial taxonomy (for both species richness or evenness) and AMR gene composition. The authors concluded that microbial communities in field soil exhibit resilience against the transposition of both free and intracellular ARGs, as well as against the introduction of microbial communities from manure [102]. Differences in microbial community and AMR gene composition between these studies may arise from variations in manure treatment and field-soil conditions, which warrant further investigation.

In addition to soil, wastewater and sludge (solid waste generated from manure and other organic materials) in the dairy environment can also contribute significantly to ARG propagation [103]. Any antimicrobial-resistant bacteria or free ARGs present in dairy environments, including bedding material or silage at any stage, could be transferred into the wastewater system [103] or the natural watershed. These free and intracellular ARGs will be accumulated in sediments through sedimentation and eventually find their way into the groundwater, surface water, and reclaimed water, and will be returned to humans through vegetable and animal products [16]. In a recent metagenomics study of dairy wastewater and soil in China, dairy wastewater contained a significantly higher abundance of free and intracellular ARGs compared to the soil [17]. The authors highlighted that the high prevalence of *tet* (X) and *tet* (X5) and *Bacteroides* as the predominant genus in feces and wastewater, which carries a large number of ARGs, may cause health threats to humans by spreading tetracycline resistance [17].

ARGs may be acquired by bacteria in dairy environments, including bedding and silage, through HGT, or mutation in the bacterial genome [18,104]. While mutations can confer resistance to antimicrobial agents in individual bacterial cells, they do not significantly contribute to the spread of AMR across bacterial populations until the antibiotic is present to impose a selective pressure. However, it is important to note that, in the clinical setting, particularly during the treatment of diseases, mutations can play a significant role in the development and dissemination of antibiotic resistance. The selective pressure exerted by antibiotic usage in clinical contexts can promote the survival and proliferation of bacteria with mutations, allowing them to thrive and potentially spread within a population [105]. On the other hand, HGT, through transformation, transduction, and conjugation, can play a significant role in the acquisition and dissemination of ARGs. The transfer of resistance genes between different species and genera through HGT is facilitated by elements such as conjugative plasmids, transposons, integrons, and bacteriophages [106,107].

During silage fermentation, microbes from starters or contaminated crops could proliferate and eventually die, potentially releasing intracellular genetic material, including ARGs, into the silage environment. However, it is important to note that the specific mechanisms of AMR gene release and transfer in silage within the context of dairy environments lack direct evidence in the current literature. While the understanding of these processes is still evolving, studies dedicated to dairy bedding and silage are notably scarce. In the broader context of AMR gene transfer, a recent study conducted in the dairy industry [108] focused on *Bacillus* strains isolated from pasteurized milk. The study revealed that, out of 114 strains, 20 were found to contain acquired resistance genes for antibiotics such as trimethoprim–sulfamethoxazole, clindamycin, erythromycin, and tetracycline [108]. The researchers examined whether these genes can be transferred between various species of *Bacillus* and discovered that the *tetL* gene from *B. cereus* BA117, which is associated with tetracycline resistance, was found to be transferable to other bacteria, such as *Bacillus invictae* BA142, *Bacillus safensis* BA143, and *Bacillus licheniformis* BA130 [108]. In conclusion, the authors warned that *Bacillus* strains with acquired AMR that are present in dairy products could pose a risk of transmitting resistance to other pathogens through HGT. However, the study focused on intra-genus transfer, and no data on extra-genus transfer is available, leaving such transfer as a hypothesis. Although this study was not directly conducted on bedding or silage, it offers insights into the potential transfer of intracellular ARGs among the bacteria associated with dairy products. A recent study [104] investigated the prevalence of acquired ARGs (intracellular) in 65 non-commercial strains of LAB to screen for the safety of potential silage inoculants. Acquired ARGs were found in 15 strains from quite a number of species, including *L. plantarum*, *L. buchneri*, *Limosilactobacillus fermentum*, *Lacticaseibacillus rhamnosus*, *Ligilactobacillus salivarius*, *Ligilactobacillus agilis*, *Lactobacillus acidophilus*, *Lactobacillus johnsonii*, *Lactobacillus diolivorans*, *Pediococcus pentosaceus*, *Enterococcus durans*, and *Enterococcus faecium* [104]. They highlighted that these acquired ARGs might be transferred to other bacteria via HGT. To evaluate the safety of practices in silage production and minimize the prevalence of ARGs, targeted investigations could assess the presence of ARGs among local bacteria before fermentation. By selectively testing bacterial strains and evaluating their potential for antibiotic resistance, the application of safe bacterial starters can be optimized, leading to improved silage safety with regard to ARG prevalence.

The same principle would apply to dairy bedding, which is naturally contaminated with mastitis pathogens or a high number of spore formers [109]. The presence of animal feces on bedding and plants has the potential to facilitate genetic material exchange, including free and intracellular ARGs, between the local bacteria and the gut bacteria, resulting in the transfer and dissemination of antibiotic resistance [110]. It is important to consider the risks associated with disease-causing bacteria, such as *S. agalactiae*, *S. aureus,* and numerous serotypes of *E. coli*, and their acquisition of ARGs through contact with compatible species of bacteria for DNA transfer, considering the species barriers and other defense mechanisms [111]. *E. coli* pathotypes can persist in feces and contaminate the environment. This contamination can lead to infections in other cows [112]. Spore formers that acquire ARGs are heat-resistant bacteria and will survive the journey from the bedding to the udder to contaminate the milk, survive processing, and then enter the human gut, where the exchange of genetic material may occur with human gut microbiota [13]. This concept is particularly relevant in the context of the gut, where research has shown that episodes of gut inflammation can result in the coexistence of pathogenic *Salmonella* and commensal *E. coli* at high densities [113]. Under these conditions, the efficient transfer of plasmids, such as the colicin-plasmid p2, occurs between these bacteria via conjugation. This finding underscores the significance of understanding HGT within the gut, as it may lead to the dissemination of AMR [113].

## 7. Critical Surveillance and Practical Management Needs

AMR surveillance systems aim to detect the changes and trends in drug-resistant microorganisms and genes and then take appropriate actions to control them [3]. Many countries have developed and implemented AMR surveillance systems for humans and animals to address the global problem of AMR. For example, beta-lactams and tetracyclines were commonly used to treat the bacterial infections of dairy cattle in the USA. Antibiotic residues, antimicrobial-resistant bacteria, and free genes can be easily found on dairy farms, contributing to the dissemination of ARGs (Table 1). Implementation of a surveillance system of dairy manure was carried out in the USA to monitor AMR development and propagation on the farm, giving a good indicator for the further adoption of management practices [114]. The outcomes emphasized recommendations to determine the AMR levels in the dairy agroecosystem and test manure management systems for their ability to degrade antibiotic residues and control antimicrobial-resistant bacteria and ARGs [114]. The study highlighted the importance of normalizing concentration data on a mass basis and conducting supportive experimental work under on-farm conditions. The collaborative efforts sought to understand on-farm exposure routes, risks, and environmental pathways of AMR spread, identifying farm practices to limit dissemination. Stakeholder involvement was crucial for translating scientific findings into practical on-farm management decisions, addressing the global antibiotic resistance crisis effectively [114]. The WHO’s Global Antimicrobial Resistance has also implemented a surveillance system for AMR in bacteria causing common human infections worldwide [115]. In Canada, the Canadian Integrated Program for Antimicrobial Resistance (CIPARS) has launched on-farm surveillance systems for food-producing animals [116]. It has the following components: (1) a herd-level antimicrobial use quantification system; (2) annually administered risk-factor questionnaires; and (3) methods for herd-level detection of AMR in three pathogens recovered from the feces and milk [116]. However, dairy environments, such as bedding and silage, have not yet been integrated into this project. The program is focused on identifying AMR in *E. coli*, *Campylobacter* spp., and *Salmonella* spp. through a culture-dependent technique as indicators of some of the problematic pathogens at key transfer points [116]. The CIPARS focuses on identifying AMR in these pathogens, as they are significant indicators of AMR in food-producing animals [116]. They are commonly associated with foodborne illnesses and are known to be major sources of resistance genes. By monitoring these specific pathogens, the program can effectively assess and manage AMR risks at critical transfer points in the food production chain [116]. Culture-independent techniques could be included to monitor the abundance of free and intracellular ARGs in dead bacteria. Evaluation of the risk of AMR gene dissemination could be facilitated by including wastewater, silage, and bedding in specific situations of high risk, as determined by the annual risk-factor questionnaires [116]. The establishment of a comprehensive monitoring program for dairy environments would be necessary to investigate multiple factors, such as the selection of appropriate target bacterial strains containing ARGs, sampling procedures, isolation and susceptibility testing methods, data recording, computing, and reporting [116]. In dairy farming, antimicrobial stewardship is essential for the judicious use of antibiotics, especially when treating preweaning calves for common respiratory and diarrheal diseases. Uyama et al. (2022) [40] emphasized that, while many Canadian dairy producers administer antimicrobials for these issues, having a veterinarian-assisted written treatment protocol greatly influences their treatment decisions [40]. Producers following such protocols are three to seven times more likely to base their treatments on a comprehensive assessment of disease symptoms [40]. Several clinical scoring systems exist to assist in diagnosing and deciding on treatments. These tools not only help in reducing overall antibiotic usage but also maintain calf health. Monitoring and surveillance programs and methodologies differ between countries and are influenced by agricultural practices, monitoring needs, and availability of guidelines. With the information on AMR in bedding and silage, a complete portrait of AMR transfer in dairy environments can be achieved, providing data for science-based recommendations for improving management practices.

Despite growing concerns about AMR, it is important to recognize that AMR is not universal and does not affect all pathogens equally. In fact, although particular pathogens/bacteria are progressively developing resistance to antibiotics that are used therapeutically, it should not be assumed that all antibiotics abruptly lose effectiveness against all bacteria [117]. Based on the level of concern for human health, Kadri et al. [118] have classified AMR under three categories, including serious, concerning, or urgent. For instance, carbapenem-resistant bacteria are considered urgent, as carbapenem is a “last-resort” antibiotic for treating infections with multi-drug resistance. The advent of carbapenem-resistant bacteria poses major concerns to public health [119,120]. Increasing resistance to third-generation cephalosporins, carbapenems, and last-resort antibiotics has caused the WHO to invest in further research into developing novel drugs/molecules discovery against priority-resistant pathogens [119,120]. To mitigate this issue, several countries have banned or restricted the use of certain antibiotics in animal husbandry [5]. For example, the European Union has prohibited the use of antibiotics as a means of promoting growth in animals since 2006, and the use of several antibiotics, including colistin, specific cephalosporins, and fluoroquinolones, has been restricted for animal use [5]. In North America, the FDA and Health Canada have taken steps to phase out the use of antibiotics for growth promotion and have implemented regulations to ensure that antibiotics are only used for therapeutic purposes under the supervision of a licensed veterinarian [121,122]. Veterinarians play a crucial role in guiding decisions related to antibiotic treatments. It is essential for these professionals to evaluate disease signs, accurately diagnose infections, and prescribe suitable treatments tailored to the individual needs of the animal [40,123]. Furthermore, antibiotic classification systems have been established to guide healthcare professionals in making informed choices and practicing antibiotic stewardship. These classification systems categorize antibiotics based on their spectrum of activity, mechanism of action, and importance in human and veterinary medicine [124,125]. By classifying antibiotics, healthcare providers can better understand their effectiveness, potential risks, and appropriate usage [126]. Such classifications facilitate the development of guidelines and protocols that promote responsible antibiotic use, support antimicrobial stewardship efforts, and help mitigate the emergence and spread of antimicrobial resistance [124,125]. In February 2019, Canada introduced a new regulation that limits the use of antimicrobials categorized as very important for human medicine in all animals used for food production. These antimicrobials, called Category 1 antimicrobials in Canada, primarily include third- and fourth-generation cephalosporins, polymyxins, and fluoroquinolones in animal farming [126]. In addition, the Canadian dairy sector established a compulsory nationwide quality assurance initiative called proAction (https://www.dairyfarmers.ca/proaction, accessed on 12 February 2024). This program mandates the maintenance of farm records concerning medical treatments, including antimicrobials. In order to efficiently address the public health issue of AMR, mitigating strategies require understanding the sources and mechanisms of transfer of resistance and applying an organized approach with the help of microbiologists, physicians, pharmacists, veterinarians, patients, and farmers [127,128].

**Figure 3:**
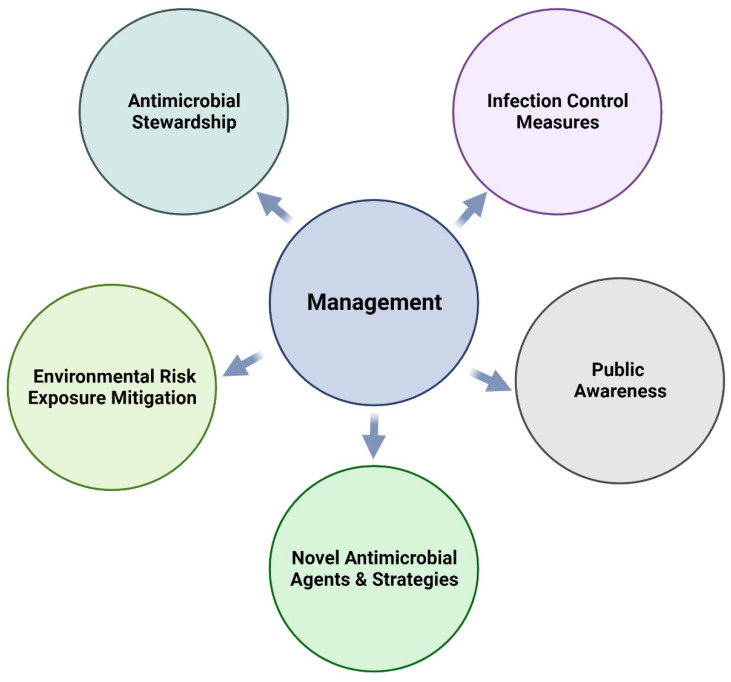
Summary of surveillance and practical management measures [116,132].

In general, the following measures can be taken to prevent the emergence and spread of AMR worldwide (Figure 3). (1) rational use of antibiotics in all settings: the appropriate use of antibiotics may be challenging for farmers and veterinarians because of their limited knowledge of the topic and the economic incentives to maintain herd health and productivity. (2) action measures that aim to prevent the spread of infection: this can be difficult due to the close contact between animals, which can facilitate the transmission of resistant bacteria and the lack of effective biosecurity measures on some farms. (3) development of strategies to mitigate the risks of environmental exposure: the complex nature of the farm environment can make it challenging to identify and control sources of AMR. Additionally, because farms are dynamic environments with many different factors that can affect the spread of bacteria, it can be challenging to design effective strategies that can address all potential sources of AMR. (4) development of rapid diagnostic tests: conducting such tests on the farm can be difficult because of their expensive nature and the requirement for specialized equipment and expertise. (5) research on AMR prevention, surveillance, and the development of novel antimicrobial agents: research in this area can be complex due to limited resources and the necessity for collaboration and coordination among researchers, farmers, veterinarians, and government agencies. (6) public awareness of antibiotic use and the risk of increasing resistance [127,128]: effective communication strategies targeted at key groups, such as farmers, veterinarians, and consumers, are key. Increasing public awareness can lead to better-informed decisions by farmers regarding antibiotic use, the adoption of responsible practices, and overall improved stewardship [129]. Educating stakeholders on the consequences of antibiotic misuse and promoting responsible use in the dairy industry can contribute to the prevention of AMR spread [129]. Unfortunately, there are few studies on the practical management of AMR reduction in bedding and silage. Novel strategies, such as feeds containing biocontrol agents, bioactive molecules, synbiotics, bacteriocins, or phage therapy that are currently used in dairy animals, may be applied to prevent the dissemination of AMR in dairy bedding and silage [130,131,132,133]. For instance, the use of dietary antimicrobial supplements, such as probiotics, prebiotics, and synbiotics, has garnered significant attention for improving feed safety and enhancing rumen health [134]. With the advent of new AMR strains, probiotics and protective cultures are gaining popularity in both the medical and livestock sectors. They may produce bacteriocins (ribosomally synthesized bacterial antimicrobial peptides (AMPs)) that can kill or inhibit closely related bacterial strains and can be considered as an alternative way to treat animal diseases, such as mastitis [135]. These promising approaches need to be investigated for bedding and silage to determine their actual effectiveness in reducing AMR on dairy farms. Bacteriophages could effectively reduce AMR by specifically targeting and killing antimicrobial-resistant bacteria. These viruses infect bacterial cells, ultimately leading to the death of the infected bacteria. By targeting specific bacterial strains or species, bacteriophages can help reduce the prevalence and spread of AMR [136]. Several studies have demonstrated the potential of bacteriophages to control mastitis-related pathogens, such as *S. aureus* [137,138]. However, the literature lacks a comprehensive study to fully understand the efficacy, safety, and practicality of bacteriophages to combat AMR on dairy farms. On the other hand, bioactive molecules, such as vitamins, minerals, amino acids, and other nutrients, can be added to animal feed to promote animal health and reduce the need for antibiotics. By improving animal health, bioactive molecules can reduce the need for antibiotics, reducing the risk of AMR development. However, studies need to be conducted to prove the efficacy of these molecules in reducing AMR dissemination.

A combination of suggested measures and the targeted use of antibiotics may pave the way to decreasing AMR prevalence. Several studies have reported the positive effect of the reduction of antibiotic use on AMR [139,140]. A comprehensive study in the Netherlands investigated whether reducing antibiotic use in livestock could decrease AMR in *E. coli* over time [140]. The researchers analyzed AMR data during the period when the Netherlands implemented a nationwide program to reduce antibiotic use in livestock. The study found a significant decrease in antibiotic-resistant *E. coli* in the pig and veal calf production sectors, and the researchers concluded that the reduction in antibiotic use was associated with a decrease in AMR in livestock and that this decrease was observed across different production systems and regions in the Netherlands [140]. The authors reported that the prevalence of resistance to older antibiotics, such as penicillins and tetracyclines, was less influenced by drug-use changes over time compared to newer and less-used antibiotics, such as third-/fourth-generation cephalosporins and fluoroquinolones [140]. Moreover, there is an urgent need for global, multidisciplinary, and long-term approaches to develop novel diagnostics and identify critical control points for effectively addressing AMR [141]. These comprehensive efforts are essential to complement the reduction in antibiotic use and the surveillance measures discussed in the previous findings. By implementing robust diagnostic tools and identifying key control points in antimicrobial use, we can enhance our ability to detect, prevent, and manage AMR across production systems. This integrated approach will contribute to the overall goal of preserving the effectiveness of antimicrobial agents and mitigating the impact of AMR on both animal and human health.

In conclusion, this review underscores the complex pathways through which AMR can propagate in dairy environments, emphasizing the need for focused research. Addressing the gaps in knowledge related to AMR prevalence in bedding and silage, and integrating these aspects into existing surveillance systems, is essential. Future investigations should prioritize the efficacy of biocontrol agents, explore the impact of reduced antibiotic use, and foster global collaborations to develop holistic strategies. These endeavors will contribute to a more comprehensive understanding of AMR dynamics in dairy settings, supporting evidence-based interventions and safeguarding both animal and human health.

## Figures and Tables

**Table 1:** Studies conducted on AMR in bedding and silage: genes, resistance classes, analysis type, geography, and microbial species.

Type	Antimicrobial Classes	Microorganisms	Genes	Analysis Type	Country	References
Bedding						
NI *	Beta-lactams, lincosamide, pleuromutilin	*B. cereus*	NI	Culture dependent (disc diffusion) and Culture independent (PCR)	China	[36]
NI	Beta-lactams, cephalosporin sulfonamide, and aminoglycoside	*K. pneumoniae*	*blaTEM*, *blaSHV*, *strA*, *strB*, *aadA1*, and *aac(60)-Ib-cr*	Culture dependent (microbroth dilution) and Culture independent (PCR)	China	[19]
Straw	Beta-lactams, fluoroquinolone, sulfonamide, aminoglycoside, macrolide, and carbapenem	*L. monocytogenes*	NI	Culture dependent (disc diffusion)	Egypt	[18]
Recycled Sand	Beta-lactams, amphenicol, aminoglycoside, sulfonamides, and tetracycline	*S. enterica* serovar Newport	*CMY-2*	Culture dependent (disc diffusion) and Culture independent (PCR)	USA	[34]
Silage						
Alfalfa	aminoglycoside and diaminopyrimidine	NA **	*aadA2*, *ant(6)-Ia*, *ant(9)-Ia*, *aph(3’)-IIa*, *aph(3’)-IIIa*, *dfrG*	Culture independent (shotgun metagenome)	China	[31]
Alfalfa	vancomycin, aminoglycoside, tetracycline, and fosmidomycin	NA	*arlR*, *emrB-qacA*, *cpxR*, and *penA*	Culture independent (shotgun metagenome)	China	[38]
Alfalfa	oxazolidinone, tetracycline, polypeptide, and amphenicol	NA	*macB*, *optrA*, *tetA*, *bcrA, efrA*, *patB, tetM* and *tetW*	Culture independent (shotgun metagenome and 16S metabarcoding)	China	[15]
Corn	fluoroquinolone, beta-lactam, aminoglycoside, polypeptide, and fosfomycin	NA	*tetW.N.W*, *tetT*, *tetA46*, *tlrC*, and *erm41*	Culture independent (shotgun metagenome)	China	[39]

* NI = not indicated; ** NA = not applicable.

## Data Availability

Not applicable.
